# Progress in EBV Vaccines

**DOI:** 10.3389/fonc.2019.00104

**Published:** 2019-02-25

**Authors:** Dwain G. van Zyl, Josef Mautner, Henri-Jacques Delecluse

**Affiliations:** ^1^German Cancer Research Center (DKFZ), Heidelberg, Germany; ^2^Institut National de la Santé et de la Recherche Médicale, Heidelberg, Germany; ^3^German Center for Infection Research (DZIF), Heidelberg, Germany; ^4^Children's Hospital, Technische Universität München, and Helmholtz Zentrum München, Bavaria, Germany

**Keywords:** γ-herpesvirus, EBV (Epstein-Barr virus), lympho proliferative disorder, oncogenic, vaccine

## Abstract

The Epstein-Barr virus (EBV) is a ubiquitous pathogen that imparts a significant burden of disease on the human population. EBV is the primary cause of infectious mononucleosis and is etiologically linked to the development of numerous malignancies. In recent years, evidence has also been amassed that strongly implicate EBV in the development of several autoimmune diseases, including multiple sclerosis. Prophylactic and therapeutic vaccination has been touted as a possible means of preventing EBV infection and controlling EBV-associated diseases. However, despite several decades of research, no licensed EBV vaccine is available. The majority of EBV vaccination studies over the last two decades have focused on the major envelope protein gp350, culminating in a phase II clinical trial that showed soluble gp350 reduced the incidence of IM, although it was unable to protect against EBV infection. Recently, novel vaccine candidates with increased structural complexity and antigenic content have been developed. The ability of next generation vaccines to safeguard against B-cell and epithelial cell infection, as well as to target infected cells during all phases of infection, is likely to decrease the negative impact of EBV infection on the human population.

## Introduction

The Epstein-Barr virus (EBV) is an oncogenic γ-herpesvirus that is endemic in human populations worldwide (1). The oncogenic potential of EBV was first suggested through its association with Burkitt lymphoma (2) and by its ability to transform B cells *in vitro* (3, 4). It is now understood that EBV is able to transform cells through the expression of Epstein-Barr nuclear antigens (EBNA) that are endowed with transactivating properties and the latent membrane proteins (LMP) that provide proliferative and survival signals (5). These proteins are expressed during non-productive (latent) infection along with several viral microRNAs (6). EBV-infected cells are also capable of supporting productive (lytic) infection, which also contributes to the development of malignancies (7–9) and is characterized by the expression of more than 80 viral genes (10) and enables the production of infectious progeny.

EBV predominantly spreads via saliva and EBV virions target epithelial cells and B cells of the oropharynx upon entering new hosts (11). Primary EBV infection usually occurs during early childhood and is not accompanied by any overt signs or symptoms. However, when EBV is acquired during adolescence or adulthood, it commonly results in infectious mononucleosis (IM) (12), a self-limiting disease whose clinical features include pharyngitis, cervical lymphadenopathy, fatigue, and fever (13). Most individuals recover from IM within a couple of weeks, but a notable portion of individuals experience fatigue that lasts for 2–6 months (14). Thus, IM is directly connected to a significant reduction in quality of life and imposes a sizable financial burden on wider society. Additionally, the occurrence of IM has been linked to an increased risk for the development of Hodgkin lymphoma (HL) (15) and multiple sclerosis (MS) (16). This suggests that a prophylactic vaccine against EBV able to prevent IM could potentially decrease the disease burden associated with HL and MS. The development of an EBV vaccine is further encouraged by the association of EBV with several other malignancies of hematopoietic or epithelial origin (17). Moreover, since EBV causes post-transplant lymphoproliferative disease (PTLD) in immunosuppressed hosts, it suggests that vaccination against EBV might be useful in hematopoietic stem cell or solid organ transplant candidates, if possible prior to transplantation (18).

## Immunogenicity of EBV Throughout its Lifecycle

**Figure 1:**
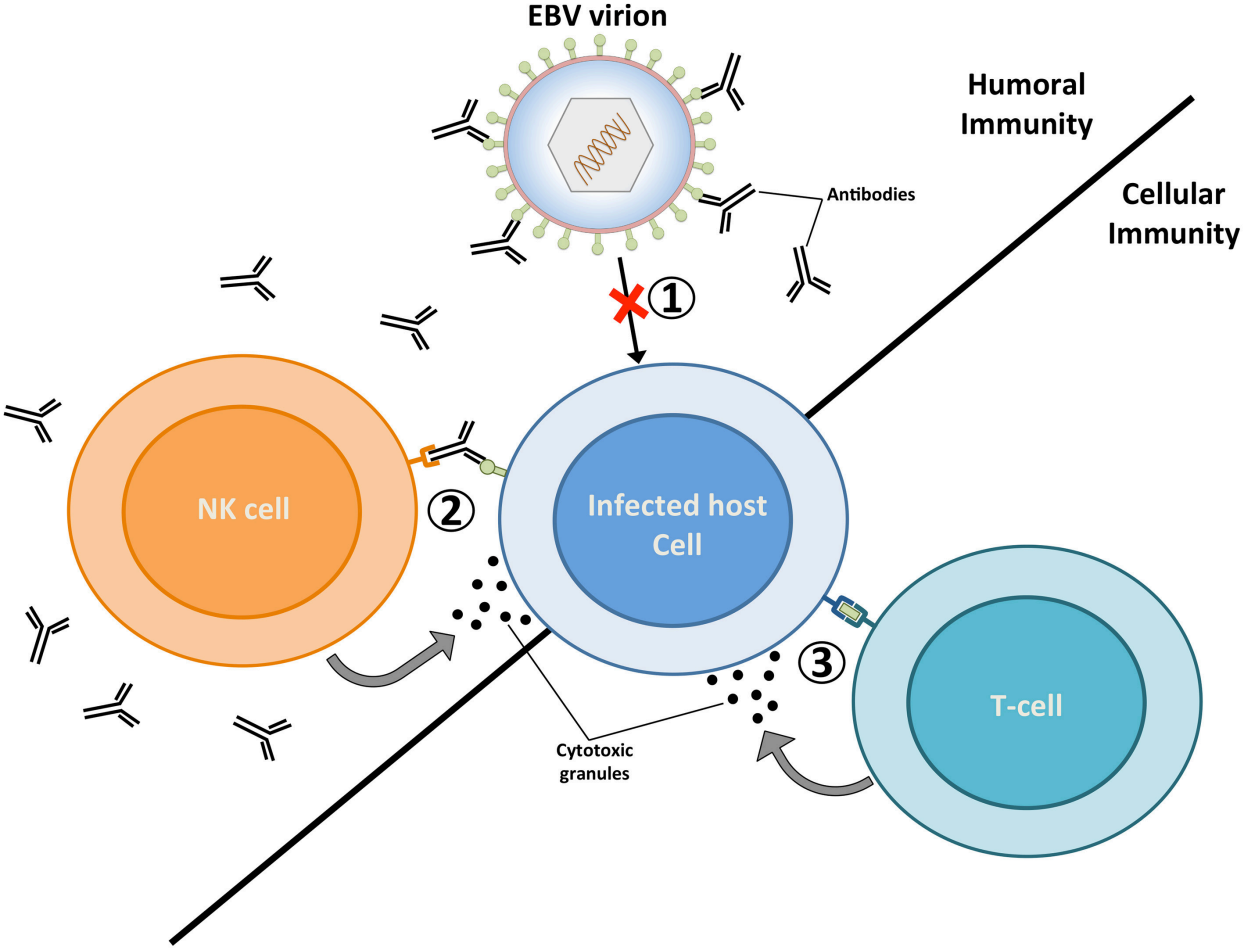
The targeting of EBV virions and EBV-infected cells by the adaptive immune system. Humoral immunity respectively targets EBV virions and EBV-infected cells through neutralizing antibodies (1) and antibody dependent-cellular cytotoxicity (ADCC) (2). The targeting of virions by neutralizing antibodies prevents the infection of host cells, while the binding of antibodies to glycoproteins at the surface of lytically replicating cells enable their recognition and elimination by natural killer (NK) cells. Vaccines geared toward stimulating humoral immunity against the major envelope glycoprotein gp350 have previously been tested in several clinical studies (20–22). (3) EBV-infected cells that display viral antigens on major histocompatibility (MHC) molecules are recognized by cytolytic T cells, which release cytotoxic granules (e.g., perforin and granzymes) and trigger apoptosis in infected cells. A vaccine that elicits EBNA3A-specific T cells responses has previously been investigated in a clinical trial (23). The ability of future EBV vaccines to stimulate potent humoral and cellular immune responses are likely to provide optimal protection against EBV infection.

**Figure 2:**
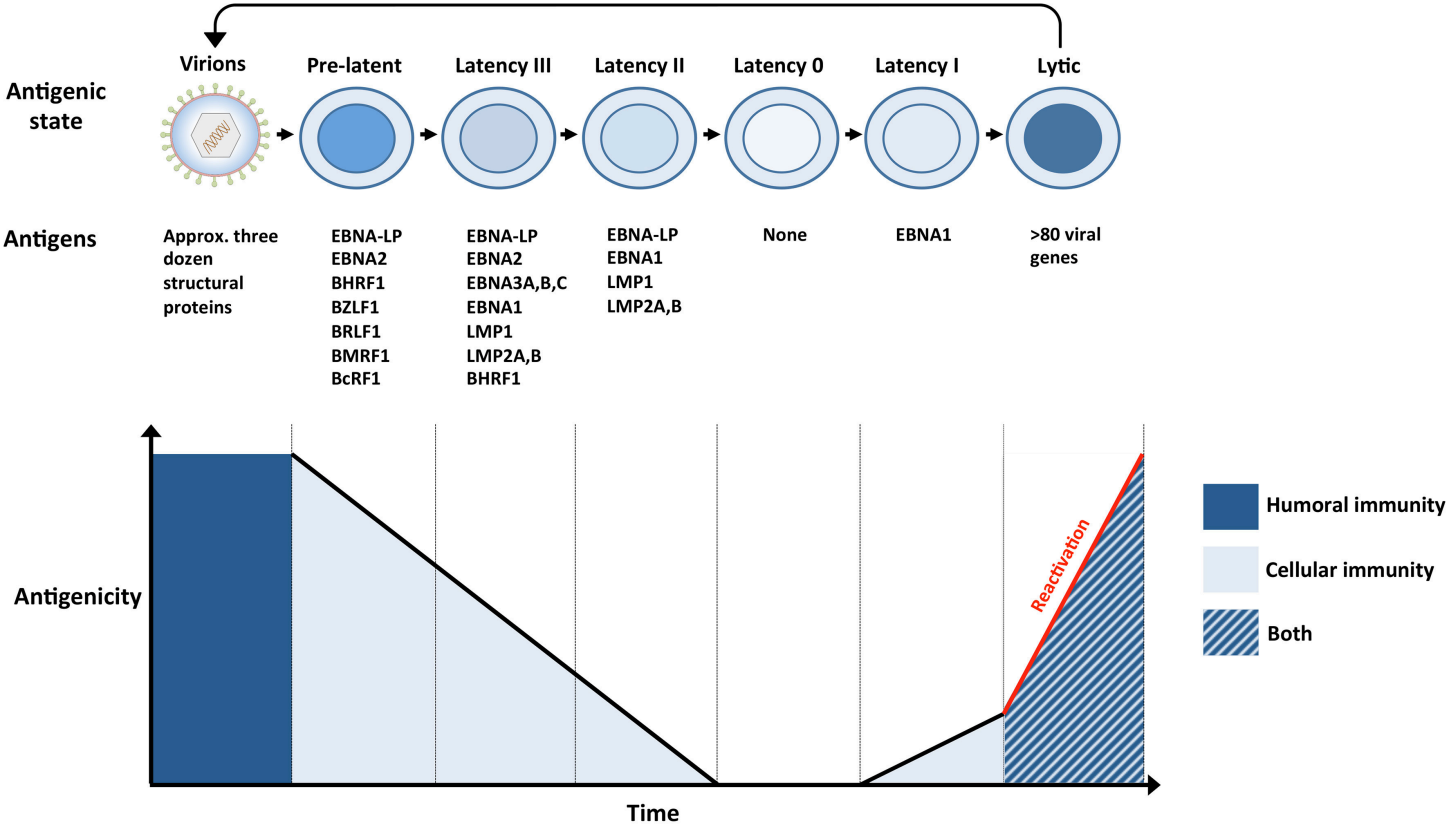
The antigenicity of EBV during a single infection cycle. **(Top panel)** Incoming virions target permissive cells within the oropharynx, lytic replication ensues and amplifies the number of virions within newly infected hosts (not shown). Virions subsequently infect naïve B cells within underlying lymphoid tissues. The presence of numerous glycoproteins at the surface of virions renders them vulnerable to neutralizing bodies (humoral immunity). Newly infected naïve B cells, also referred to as pre-latent, express a handful of lytic and latent antigens. Infected B cells subsequently transition through several latency stages (III → II → 0), gradually reducing the number of EBV antigens that are expressed, eventually resulting in the establishment of latency 0 in quiescent memory B cells. Since no EBV antigens are expressed during latency 0, it enables infected cells to evade immune recognition. However, EBV-infected memory B cells are maintained through normal B-cell homeostatic mechanisms and express EBNA1 when they divide (latency I). The expression of viral antigens during pre-latency, latency I, II, and III renders the infected cells vulnerable to EBV-specific T cells (cellular immunity). Circulating B cells that re-enter the nasopharyngeal lymphoid system differentiate into plasma cells that support lytic replication. The expression of approximately 80 viral proteins, several of which are displayed at the surface of infected cells, exposes these cells to EBV-specific T cells (cellular immunity), and ADCC (humoral immunity). Virions released from lytically replicating cells can initiate another cycle of infection if they are not targeted by neutralizing antibodies (humoral immunity). **(Bottom panel)** The number and nature of EBV antigens fluctuates throughout a single infection cycle and these proteins are targeted by humoral immunity and/or cellular immunity.

EBV infection and the ensuing lifelong persistence is a complex, multistep process that starts with the infection of permissive cells within the oropharynx, and culminates in the maintenance of EBV in circulating memory B cells (19). The germinal center model (GCM) of EBV persistence suggests that EBV utilizes the normal pathway of B-cell differentiation to achieve this feat. Since EBV is capable of implementing various latency and lytic transcription programs, it suggests that EBV assumes distinct antigenic states within infected individuals (Table 1). Moreover, since the nature of these antigens varies, they offer unique challenges to the adaptive immune system (Figure 1). Yet, despite the wide variety of antigens that predominate throughout the EBV life cycle, EBV vaccines candidates have traditionally only focused on a limited number of EBV antigens (See the review by Cohen (24) for a summary on these vaccine candidates. We now consider the various antigenic states of EBV during a single infection cycle and how vaccination may aid their recognition and elimination (Figure 2).

**Table 1:** Different EBV transcription programs and associated diseases.

**Transcription program**	**Expressed proteins**	**Description or function**	**Associated disorder**
Pre-latency	BZLF1, BRFL1, BMRF1, BcRF1 EBNA2, BHRF1, EBNA-LP	The expression of various latent and lytic proteins improves survival and immune evasion of newly infected B cells.	
Latency III	EBNA−1, −2, −3A, −3B, −3C and -LP LMP−1,−2A and LMP-2B	Expression of the full complement of latent proteins serves to activate naïve B cells and leads to their proliferation as B-cell blasts.	PTLD
Latency II	EBNA1, LMP1, LMP2A	Mimics T-cell help and BCR signaling so that GC B cells are rescued into the memory compartment.	Hodgkin lymphoma, Nasopharyngeal carcinoma
Latency I	EBNA1	The expression of EBNA1 enables the viral genome to be replicated along with the host genome during memory B-cell homeostasis.	Burkitt lymphoma, Gastric carcinoma
Latency 0	None	The absence of EBV antigens enables immune escape and ensures survival of long-lived memory B cells	
Lytic	More than 80 viral genes are expressed	The production of virions promotes the continued infection of permissive cells within the same host and enables the horizontal transfer of virions to other individuals.	

### EBV Virions

EBV virions are large, multilayered particles that comprise numerous viral proteins. A single EBV virion comprises more than 30 different capsid, tegument, and envelope proteins (25). Of these various virion components, it is the envelope glycoproteins that mediate the initial stages of infection in permissive cells. Whilst EBV has been reported to infect various cell types within its host, only details regarding B-cell and epithelial cell infection are known. A total of five envelope glycoproteins (*viz* gp350, gp42, gH, gL, and gB) are used by incoming virions to gain access to the cytosol of epithelial cells and B cells. EBV virions bind to CD21 (26) or CD35 (27) on B cells through the use of gp350, after which gp42, in complex with gH and gL (28), interacts with HLA class II molecules to trigger gB-mediated viral-host membrane fusion within endosomes (29). In contrast, epithelial cell infection relies on the interaction between BMRF2 and integrins (β1 family and α5β1) (30) followed by the interaction of gH/gL with ephrin receptor A2 (EphA2) (31, 32) and integrins (viz. α*vβ*5, α*vβ*6, α*vβ*8) (33, 34) to trigger gB-mediated fusion at the plasma membrane. Since these envelope proteins play such a crucial role during the early phase of infection, they are excellent vaccine targets (34–39). The ability of sera from EBV-positive individuals to block infection augurs well for the development of a vaccine that induces neutralizing antibodies against EBV glycoproteins (40). However, since EBV uses a different set of glycoproteins to infect B cells and epithelial cells, it is unclear whether EBV vaccines would have to target multiple glycoproteins to efficiently block EBV infection in these cell types. The isolation of a gH/gL-specific antibody that blocks B-cell and epithelial cell infection suggests that vaccination with gH/gL alone might be sufficient to prevent B-cell and epithelial cells infection (39). However, it is unclear whether vaccine-induced anti-gH/gL antibodies would be capable of the same feat. The prospect of blocking EBV infection with neutralizing antibodies is further complicated when one considers that EBV-specific antibodies have even been shown enhance epithelial cell infection (38). Moreover, EBV is also capable of infecting T cells (41, 42) and NK cells (43) through yet undefined mechanism. This suggests that EBV vaccines cannot at present be rationally designed to prevent the infection of all susceptible cell types. Lastly, it is also unknown whether vaccination can induce sufficient levels of neutralizing antibody within the oropharynx to prevent EBV infection. Since the majority of animal models of EBV infection do not employ virus challenge via the normal infection route, they are incapable of assessing mucosal immunity (44–53).

### Pre-latent Phase

Once EBV colonizes the oropharynx, lytic replication ensues, and enables the infection of naïve B cells within underlying lymphoid tissues (54). Within the first 48 h of infection, prior to the first cell division, naïve B cells transiently express a subset of latent and lytic genes and are termed pre-latent (Table 1) (55–58). Since these cells do not express structural proteins or genes necessary for DNA replication, they do not support productive infection (59). However, since several antigens are expressed at such an early time point, it renders the infected B cells vulnerable to the cellular immune response. Indeed, recently infected B cells are recognized to varying degrees by latent protein- (e.g., EBNA2 and EBNA-LP) and lytic protein- (e.g., BHRF1) specific CD4^+^ and CD8^+^ T cells (60). However, of the various epitopes displayed by pre-latent B cells, EBNA2 MHC-I-restricted epitopes are most efficiently recognized at an early time point and this marks EBNA2 as a promising vaccine target. Pre-latent cells do not only display epitopes from *de novo* expressed antigens, but also from proteins that are associated with incoming virions. Recently infected B cells are well-recognized by envelope- (e.g., gp350, gH, gB) and tegument- (e.g., BNRF1) specific CD4^+^ T cells (60–63). Thus, structural proteins are not only recognized by neutralizing antibodies, but also by T-cell responses. Therefore, vaccines that comprise structural antigens could potentially elicit protective T-cell responses in addition to generating neutralizing antibodies, enabling the targeting of virions and of recently infected cells at a very early time point after infection.

### Latent Phase

The expression of lytic and latent proteins during pre-latency is short lived and is succeeded by a series of latency transcriptional programs. The sequential implementation of latency transcription programs drive naïve EBV-infected B cells to proliferate (latency III), undergo a GC reaction (latency II) and differentiate into quiescent memory B cells (latency 0) that occasionally express EBNA1 (latency I) (54) (Figure 2). Since healthy EBV-positive individuals recognize multiple latent proteins (60, 64–67), vaccination with latent antigens might enable the recognition of B cells that implement latency III, II, or I. However, since no viral antigens are expressed during latency 0, quiescent memory B cells successfully evade immune recognition. The inability of the immune system to target these cells suggests that vaccine-induced sterile immunity might be very difficult to achieve against EBV. In order to prevent the establishment of latency 0 in infected B cells, vaccine-induced immunity would in principle have to efficiently target EBV virions and infected cells before their transition to latency 0. This would be especially important if EBV-infected B cells are able to directly transition to the memory phenotype (68). However, it is unclear whether sterile immunity against herpesviruses is even possible (see section Vaccination Lessons From Other Herpesviruses). Nonetheless, since latent antigens are expressed in EBV-associated diseases and malignancies, vaccination with latent proteins could reduce the disease burden of EBV. Indeed, the adoptive transfer of latent protein-specific T cells has provided a clinical benefit to a subset of patients suffering from EBV-associated malignancies (69–72). Therefore, vaccines that induce latent protein-specific responses might enable the targeting of EBV-infected cells before and after transformation.

### Lytic Infection

Lytically infected cells play a crucial role in the establishment of EBV infection, its maintenance, and the horizontal transfer of EBV between hosts (54). The production of virus during the early phase of primary infection increases the number of B cells that are infected, while lytic replication during persistent infection ensures that the pool of infected B cells is continuously replenished. Whilst epithelial cells are capable of spontaneous virus production (73), the GCM model suggest that EBV-infected B cells require terminal differentiation into plasma cells in order to support lytic replication (74). EBV-infected cells achieve the production of virions through the coordinated expression more than 80 immediate early (IE), early (E), and late (L) lytic genes (10). The expression of so many antigens by lytically replicating cells makes them subject to immune control. T cells isolated from healthy EBV-positive individuals frequently recognize IE, E, and L antigens (75–80). Moreover, since lytically replicating cells display viral glycoproteins at their surface, they can be targeted by antibody-dependent cellular cytotoxicity (ADCC) (81–84). Thus, lytically replicating cells are subjected to the cellular and adaptive immune system.

## Vaccination Lessons From Other Herpesviruses

The struggle toward a licensed prophylactic vaccine is not limited to EBV and has also been the case for the majority of human herpesviruses. The only exception to this trend is the alpha herpesvirus Varicella-zoster virus (VZV), which is responsible for the development of varicella and zoster (85, 86). VZV establishes latency after primary infection, is carried lifelong and is controlled in healthy individuals through the coordinated activities of humoral and cellular immunity (87). Vaccination against VZV is carried out with a live-attenuated virus that induces immune responses comparable to wild-type VZV infections (88). Vaccinated individuals develop humoral and cellular immune responses that target numerous VZV proteins (88–90). Vaccination against herpesviruses has also been successfully carried out in animals, with Marek's disease virus (MDV) representing an exemplary case. MDV is a highly oncogenic poultry pathogen that causes the development of lymphomas (91, 92). Vaccination against MD is also carried out with a live-attenuated virus and successfully prevents the development of tumors (93).

Whilst vaccination against VZV and MDV successfully prevents the manifestation of disease, they do not prevent infection with wild-type strains (94, 95). Similarly, vaccination against EBV might be able to reduce EBV-associated diseases and malignancies without achieving sterile immunity (96). Considering what is known about successful vaccination against VZV, it is reasonable to assume that the ideal EBV vaccine should generate immune responses that mimic those observed during wild-type infections. However, since live-attenuated herpesvirus vaccines persist in infected individuals (97), it is unlikely that EBV, an oncogenic virus, would be suitable as a live-attenuated vaccine. Considering the complexity of EBV infection, in terms of the different cell types that are infected, the ability of EBV to spread directly from cell-to-cell (30, 98) and the diverse number of antigens that predominate during the EBV life cycle, EBV vaccines have to deal with the challenging task of being efficacious and highly safe.

## Prophylactic Vaccines

**Figure 3:**
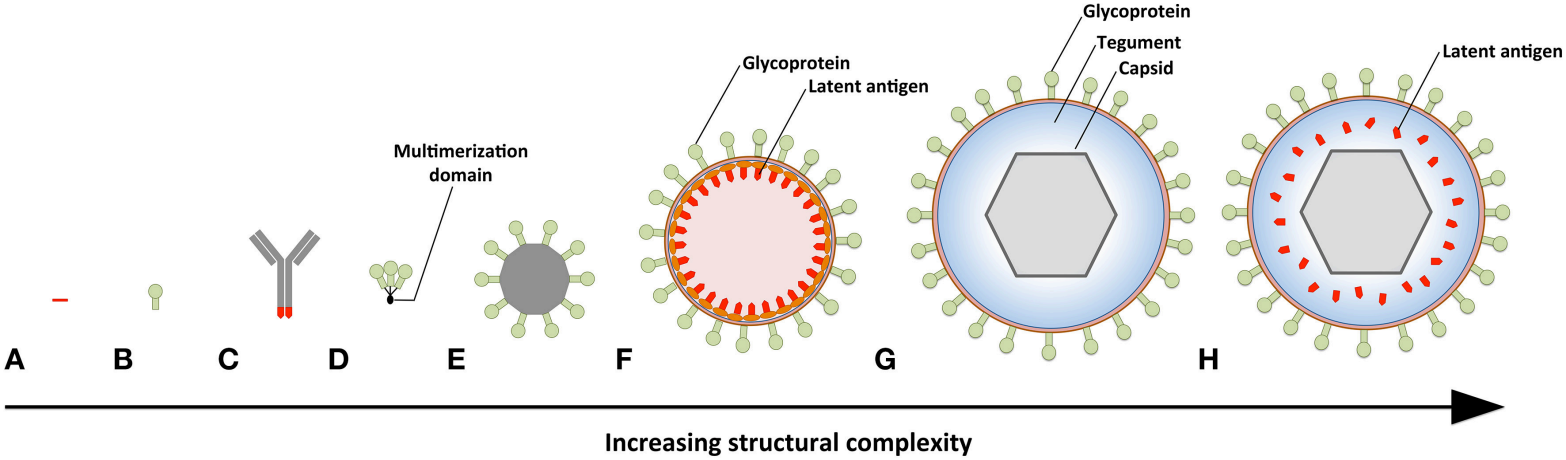
Prophylactic EBV vaccine candidates vary in their structural complexity and antigenic content. **(A)** An epitope peptide from EBNA3A. **(B)** Recombinant gp350 expressed as a monomeric protein. **(C)** EBV latent antigens (red) conjugated to dendritic cell- or B-cell-specific antibodies. **(D)** Multimeric forms of EBV structural proteins can be generated through the use of multimerization domains (black). **(E)** Self-assembling ferritin nanoparticles presenting gp350. **(F)** Chimeric NDV VLPs containing lytic (green) and latent (red) EBV antigens. Several EBV antigens (*viz*. gp350, gH/gL, g, EBNA1, and LMP2) have been targeted using this platform. **(G)** EBV VLPs comprise numerous envelope (green), tegument (blue), and capsid (gray) proteins. **(H)** The tegument of EBV VLPs can be modified to contain latent antigens (red).

The potential of vaccination to mitigate EBV-associated diseases was eluded to within a decade of EBVs discovery (99), with so-called membrane antigen being suggested as a potential target (100). This was followed by three decades of research in which gp350 was championed as the vaccine candidate of choice (24). Since gp350-specific antibodies block B-cell infection, gp350 was marked as a promising vaccine target. However, when a subunit gp350 vaccine was finally tested in a phase 2 clinical trial, it failed to prevent EBV infection despite inducing neutralizing antibodies in vaccinees (20). This was succeeded by the development of numerous vaccine platforms that vary in antigenic content and structural complexity (Figure 3, Table 2).

**Table 2:** Summary of prophylactic EBV vaccine candidates that have been developed.

**Vaccine category**	**EBV antigens included**	**Results**
Epitope vaccine	EBNA3A (23)	Vaccination induced EBNA3A-specific T-cell responses, but the vaccine did not protect against EBV infection. Vaccinated individuals had a lower incidence of IM, but sample sizes were to small to enable statistical analysis.
Antigen-antibody conjugates	EBNA1 (101)	Targeting of dendritic cells enabled the induction of EBNA1-specific CD4^+^ and CD8^+^ T cells and vaccination of humanized mice generated EBNA1-specific T cells.
	Several latent antigens (102, 103)	Multiple Antibody-antigen conjugates were several orders of magnitude more efficient than peptide at stimulating cytotoxic CD4^+^ T cells.
Monomeric	gp350 (20)	Vaccination induced neutralizing gp350-specific antibodies, reduced the incidence of IM but did not protect against EBV infection.
Multimeric	Tetrameric gp350 (104)	Rabbits vaccinated with tetrameric gp350 had neutralizing titers 19-fold higher than rabbits vaccinated with monomeric gp350.
	Trimeric gH/gL and trimeric gB (105)	Serum neutralizing titers were respectively >100-fold and 18-fold higher in rabbits vaccinated with trimeric gH/gL and trimeric gB compared to monomeric gp350.
Nanoparticles	gp350 (106)	Mice and monkeys that were vaccinated with gp350-containing nanoparticles generated potent gp350-specific neutralizing antibodies compared to animals vaccinated with monomeric gp350.
Chimeric NDV VLPs	gp350 (107)	NDV-VLPs containing gp350 elicited superior neutralizing antibodies in vaccinated mice compared to monomeric gp350.
	gH/gL, gp42, LMP2, EBNA1 (108)	The NDV VLP platform was utilized to incorporate various EBV latent and lytic antigens.
EBV VLPs	More than three dozen structural proteins (109)	EBV VLPs elicited neutralizing EBV-specific antibodies and T-cell responses in vaccinated mice
	More than three dozen structural proteins (63)	EBV VLPs stimulated structural protein-specific T cells to the same degree as wtEBV.
	More than three dozen structural proteins and EBNA1 (110)	Modified EBV VLPs stimulated structural protein- and latent protein-specific T cells and afforded increased protection in humanized mice.

The simplest vaccine platform that has been considered for EBV infection is an EBNA3A epitope peptide mixed with tetanus toxin as a water-in-oil emulsion (Figure 3A) (23). In contrast to recombinant gp350, which was developed to elicit neutralizing antibodies, the EBNA3A epitope vaccine was designed to induce EBV-specific T-cell responses that recognize infected B cells displaying latency III. Vaccination with the EBNA3A epitope peptide successfully induced the relevant CD8+ T-cell responses in ~90% of vaccinees, but failed to prevent EBV infection. Although vaccination appeared to reduce incidence of IM, the number of participants was too few for statistical analysis.

The next group of EBV vaccine candidates is based on antigen-antibody conjugates, or antigen-armed antibodies (AgAbs), which specifically deliver antigenic peptides to antigen-presenting cells (APCs) (Figure 3B) (111). αDEC-205 equipped with EBNA1 is processed by dendritic cells and enables the expansion of EBNA1-specific CD4+ and CD8+ T cells from the blood of EBV-positive individuals (101). Also, humanized mice vaccinated with αDEC-205-EBNA1 successfully developed EBNA1-specific IgM and T-cell responses. EBV latent antigens have also been fused to αCD19, αCD20, αCD21, and αCD22 to enable B-cell-mediated stimulation of EBV-specific T cells (102). Antigenic epitopes fused to B-cell-specific antibodies are up to ~4,000-fold more antigenic than peptide alone and enable the stimulation of cytolytic CD4+ T cells (102, 103). The ability of antigen-antibody conjugates to potently stimulate latent protein-specific T cells suggests that they might induce protective T-cell responses upon vaccination.

Owing to the weak immunogenicity of monomeric subunit vaccines (112–115), several groups have moved toward the development of multimeric vaccines that arrange EBV antigens in a repetitive manner. This enables multivalent, long-lasting, stimulatory interactions with the immune system, and generates considerably stronger B- and T-cell responses than monomeric proteins (116). A direct approach for increasing the immunogenicity of individual EBV antigens is through the use of multimerization domains (Figure 3C). One group has successfully generated multimeric gp350 (104) and gH/gL (105) by fusing the individual antigens to mutimerization domains (e.g., the T4 bacteriophage foldon). They demonstrated that rabbits vaccinated with the multimeric proteins developed superior neutralizing antibody responses compared to their monomeric counterparts. However, both monomeric and multimeric gH/gL were shown to outperform all other antigens, with multimeric gH/gL inducing serum neutralization titers >100-fold higher than monomeric gp350.

EBV antigens have also been introduced into virus-like particles (VLPs) (107, 108) and self-assembling nanoparticles (106). These platforms enable monomeric antigens to be displayed in a manner highly comparable to EBV virions, but since they lack viral DNA they are incapable of causing disease. One group has developed chimeric VLPs by fusing EBV antigens to Newcastle disease virus (NDV) structural proteins (Figure 3D) (107, 108). Chimeric NDV VLPs containing gp350 have been shown to induce superior neutralizing antibody responses upon vaccination in mice compared to monomeric gp350 (107). Subsequently, the NDV VLP platform was utilized to generate immunogenic particles that contained multiple EBV antigens, including envelope proteins (gB, gH/gL, gp350) and latent proteins (EBNA1 and LMP2) (108). The potential of these particles to stimulate structural protein- and latent protein-specific immune responses suggests they might enable the targeting of EBV virions and EBV-infected cells in vaccinated individuals. The gp350 antigen has also been fused to *Helicobacter pylori*-bullfrog hybrid ferritin to generate highly immunogenic self-assembling nanoparticles (Figure 3E) (106). Nanoparticles containing gp350 induced significantly higher neutralizing antibody titers in mice and monkeys in comparison to monomeric gp350. The incorporation of gp350 into ferritin nanoparticles was elegantly shown to enhance the presentation and recognition of the CD21-binding site on gp350. Moreover, mice vaccinated with the gp350 nanoparticles were successfully protected against a recombinant vaccinia virus that expressed gp350. However, since gp350 did not play a functional role in the infection process and vaccination success or failure was solely based on the weight of the animals, it is difficult to relate these findings to vaccination against EBV in humans.

Another approach to EBV vaccination is through the use of EBV VLPs. Since EBV VLPs are structurally similar to EBV and comprise numerous viral antigens (*viz.*, envelope, capsid, and tegument), they have the potential to elicit immune responses against multiple EBV antigens. By deleting genetic elements (109, 117, 118) or proteins (63) involved in DNA packaging, the generated EBV VLPs contain little to no viral DNA (Figure 3F). One group has developed a VLP-producing packaging cell line by deleting the terminal repeats (TR), involved in DNA packaging, and six viral genes that contribute to transformation or production of virions (EBNA2, EBNA3-A, -B, -C, LMP1, and BZLF1) (109). Vaccination of mice with these VLPs induced polyvalent EBV-specific humoral and cellular immune response, highlighting the potential of EBV VLPs to elicit broad immune responses. Impressively, the potency of vaccine-induced neutralizing antibodies was comparable to the anti-gp350 MAb 72A1. This finding is supported by studies that have shown UV-inactivated EBV to induce potent neutralizing antibodies (107, 108). We have developed an alternate EBV VLP packaging cell line by deleting BFLF1/BFRF1A and gB from the EBV genome (9, 63). By deleting BFLF1/BFRF1A we improved the purity of the VLPs relative to those obtained with a ΔTR genome in that they contained no detectible DNA (63), while deletion of gB prevents VLPs from fusing with host cells and greatly reduces their pathogenic potential (9, 119, 120). We subsequently increased the antigenic spectrum of EBV VLPs by fusing latent antigens to the major tegument protein BNRF1 (Figure 3G) (110). VLPs containing latent antigen are capable of stimulating both structural protein- and latent protein-specific T cells and afford increased protection against EBV infection in humanized mice. Since EBV encodes more than a dozen tegument proteins, EBV VLPs have the potential to accommodate multiple immunodominant latent antigens. By fine-tuning the antigenic cargo of EBV VLPs, they might enable the induction of immune responses that recognize viral antigens that predominate during the early stages of the infection and in latently infected cells.

## Therapeutic Vaccines

Therapeutic EBV vaccines aim at boosting and sustaining antiviral adaptive immune responses in patients with virus-associated disorders. For several reasons, most therapeutic vaccination approaches have focused on NPC. First, almost all cases of the non-keratinizing subtype of nasopharyngeal carcinoma (NPC), which represents >95% of NPC in endemic regions, are EBV-positive and consistently express EBNA1, LMP2, and to variable degrees LMP1 (121). Second, EBNA1 and LMP2 are major targets of the virus-specific T-cell response in healthy virus carriers, and CD4^+^ and CD8^+^ T cells against these antigens have been detected in tumor patients (122, 123). Moreover, virus-specific T cells can be cultured from NPC tissues suggesting that T-cell function is either maintained or not irreversibly impaired in these patients (123). Third, HLA loss is uncommon in NPC and clinical responses have been observed after infusion of EBV-specific T-cell preparations, suggesting that tumor growth can be controlled by the immune system (124, 125). Based on these findings, different therapeutic vaccines have been designed and tested in NPC patients. All of these vaccines have been well-tolerated with minimal side effects and no evidence of dose-limiting toxicity.

In the first therapeutic vaccination trial for NPC, 16 patients with residual disease received four cycles of autologous monocyte-derived dendritic cells (DC) loaded with LMP2-specific CD8^+^ T-cell epitope peptides (126). In more than half of these patients, increases in LMP2-specific CD8^+^ T cells were noted and these increases were sustained for 3 months before declining. Partial clinical responses were observed in two patients that had shown heightened virus-specific T-cell frequencies. This study used a small number of defined CD8^+^ T-cell epitope peptides selected on the basis of the patients' HLA type. In order to boost a wider range of T-cell specificities including those still undefined or presented on HLA class II molecules, autologous DC expressing truncated LMP1, and full length LMP2 protein were used (127). Partial clinical response or stable disease was achieved in three of the 16 vaccinated patients. Based on the widely used modified vaccinia Ankara (MVA) vector, a therapeutic vaccine was designed that expresses a fusion protein of full length LMP2 and the C-terminal half of EBNA1. Two dose escalation phase IA clinical trials were conducted with this MVA-EBNA1/LMP2 vaccine on NPC patients in Hong Kong and the United Kingdom (128, 129). Patients who had received the highest dose responded to EBNA1, LMP2, or both. Moreover, increased CD8^+^ and CD4^+^ T-cell responses against LMP2 and EBNA1 were observed in both patient cohorts in Hong Kong and UK, demonstrating that the vaccine was immunogenic in different ethnicities with different HLA types and EBV strain variants. In ongoing phase IB and II trials, immunogenicity, and clinical efficacy of the vaccine are further examined.

Despite these encouraging results, therapeutic vaccination alone is unlikely to reduce disease recurrence in the majority of patients. Additional studies are needed to test whether treatment outcomes can be improved by combining therapeutic vaccination with other forms of immunotherapy, e.g., adoptive T-cell therapy or checkpoint inhibition. Besides, issues of optimal combination need to be addressed for incorporating immunotherapy into standard treatment protocols.

## Conclusions and Future Prospects

The number of EBV vaccine candidates has greatly increased over the last decade. The latest advances in vaccine technology, coupled with our growing understanding of EBV biology and immunology, have enabled emerging EBV vaccine candidates to directly address the shortcomings of a monomeric gp350 vaccine. However, since the correlates of protection against EBV have not been clearly defined, it is hard to reliably predict the ideal EBV vaccine targets and whether humoral immunity or cellular immunity or both should be engaged. Vaccines comprising a limited set of EBV antigens would certainly be easier to manufacture and safer compared to vaccines that contain a large combination of EBV antigens. However, if vaccination with a limited set of antigens is unable to preclude the establishment of latency, vaccines might have to increase their antigenic spectrum to include multiple structural antigens and perhaps even latent antigens. However, since latent proteins are highly polymorphic compared to structural proteins (130, 131), it suggests that they are considerably more challenging to target across multiple geographic regions. Nevertheless, since all EBV-associated malignancies express latent antigens, it suggests that they are worth exploring as vaccine targets. Also, different vaccine types might end up having different aims and target populations. While vaccines based on a one or several antigens might be sufficient to prevent the occurrence of IM and its complications such as multiple sclerosis without necessarily being able to confer sterile immunity, a cocktail of EBV antigens from different EBV strains, carrying latent and lytic proteins might be able to achieve a higher level of protection against infection and its malignant and non-malignant consequences. The first type of vaccine would probably be indicated in immunocompetent western populations at low risk of EBV-associated tumors and the second in populations vulnerable to endemic EBV-associated malignancies or persons awaiting organ transplantation. However, only vaccination campaigns will be able to determine which types of vaccines confer protection and to what degree.

## Author Contributions

All authors have made a substantial contribution to the work. DvZ wrote the original draft and generated the graphics. JM wrote the section on therapeutic vaccines. H-JD and JM reviewed and edited the final manuscript.

### Conflict of Interest Statement

The authors declare that the research was conducted in the absence of any commercial or financial relationships that could be construed as a potential conflict of interest.
